# Adherent-invasive *Escherichia coli pduB* drives vitamin B_12_-dependent colitis in mice

**DOI:** 10.1128/mbio.01698-26

**Published:** 2026-09-16

**Authors:** Shahanshah Khan, Jungyeon Kim, Alexander Lafrance, Sydney Field, Xi Li, Lisa N. Kinch, Kevin J. Forsberg, Tamia A. Harris-Tryon, Qian Cong, Kim Orth, Hans-Christian Reinecker, Josephine Ni

**Affiliations:** 1 Division of Digestive and Liver Diseases, Department of Internal Medicine, University of Texas Southwestern Medical Center 196284, Dallas, Texas, USA; 2 Department of Molecular Biology, University of Texas Southwestern Medical Center 465751, Dallas, Texas, USA; 3 Department of Microbiology, University of Texas Southwestern Medical Center 12334, Dallas, Texas, USA; 4 Department of Dermatology, University of Texas Southwestern Medical Center 652461, Dallas, Texas, USA; 5 Eugene McDermott Center for Human Growth and Development, University of Texas Southwestern Medical Center 130372, Dallas, Texas, USA; 6 Department of Biophysics, University of Texas Southwestern Medical Center 12334, Dallas, Texas, USA; 7 Harold C. Simmons Comprehensive Cancer Center, University of Texas Southwestern Medical Center 12334, Dallas, Texas, USA; 8 Howard Hughes Medical Institute 12334, Dallas, Texas, USA; 9 Department of Biochemistry, University of Texas Southwestern Medical Center 205285, Dallas, Texas, USA; Freie Universitat Berlin, Berlin, Germany

**Keywords:** adherent-invasive *Escherichia coli*, Crohn's disease, 1,2-propanediol utilization (pdu) operon, vitamin B_12_, cell invasion, host-microbe interactions

## Abstract

Adherent-invasive *Escherichia coli* (AIEC) are strongly implicated in the pathogenesis of Crohn’s disease, but the virulence factors driving their invasive and pro-inflammatory behavior remain incompletely defined. Here, we demonstrate that vitamin B_12_ availability modulates *pdu*-dependent AIEC virulence. The 1,2-propanediol utilization (*pdu*) operon, which requires vitamin B_12_ as a cofactor, has previously been associated with bacterial virulence. Here, we validated this association and demonstrated its functional relevance in mouse models of colitis. Deletion of *pduB*, a structural component of the *pdu* microcompartment, in the prototypical AIEC strain NRG857c impaired invasion of intestinal epithelial cells without affecting bacterial growth, biofilm formation, or intestinal colonization. In contrast, *pduB* deletion reduced mucosal association and significantly attenuated colitis in *Il10^−/^*^−^ mice. Moreover, loss of *pduB* protected against AIEC-induced epithelial barrier dysfunction and reduced expression of pro-inflammatory cytokines, including *Il6*, *Il1b*, and *Tnf-a*. Unexpectedly, *pduB* deletion was associated with elevated host serum vitamin B_12_ levels, and exogenous vitamin B_12_ supplementation directly inhibited AIEC invasion *in vitro* and effectively protected *Il10^−/^*^−^ mice from AIEC-induced colitis. Furthermore, vitamin B_12_-mediated suppression of AIEC invasion and barrier dysfunction was dependent on an intact *pdu* pathway. Together, these findings establish *pduB* as a key regulator of AIEC virulence. Our findings identify vitamin B_12_ availability as a host-modifiable factor that influences AIEC pathogenicity, suggesting potential therapeutic strategies for AIEC-driven intestinal inflammation in Crohn’s disease.

**IMPORTANCE:**

In this study, we demonstrated that vitamin B_12_ availability modulates a metabolic interaction between adherent-invasive *Escherichia coli* (AIEC) and the host micronutrient vitamin B_12_ that influences AIEC epithelial invasion. We confirm that a gene within the 1,2-propanediol utilization pathway (*pduB*) is critical for AIEC invasion and the development of intestinal inflammation in multiple mouse models. We further identify that vitamin B_12_, a cofactor for metabolic reactions within the Pdu microcompartment, is altered in the host during infection, suggesting changes in the microbe-host vitamin B_12_ utilization dynamics. Functionally, we also show that vitamin B_12_ supplementation significantly inhibited AIEC invasion of epithelial cells *in vitro.* Lastly, we found that vitamin B_12_ supplementation provided strong protection against AIEC-induced colitis in *Il10^−/^*^−^ mice, preventing weight loss, rectal prolapse, tissue damage, and inflammatory infiltration, even in animals colonized with wild-type AIEC. Broadly, our work highlights vitamin B_12_ availability as a modifiable host factor that influences AIEC pathogenicity and suggests new therapeutic avenues for therapeutic strategies targeting microbe-nutrient interactions in inflammatory bowel disease.

## INTRODUCTION

Inflammatory bowel disease (IBD), including ulcerative colitis (UC) and Crohn’s disease (CD), affects between 2 and 3 million patients in the United States and accounts for nearly $8.5 billion in U.S. healthcare costs annually (1). The etiology, progression, and clinical outcomes of IBD depend on interactions between a myriad of genetic, environmental, nutritional, and microbial factors (2–4). Among these variables, micronutrient deficiencies and the expansion of pathobiont adherent-invasive *Escherichia coli* (AIEC) in the gut microbiome have both individually been associated with IBD; however, the relationship between them and its implications for IBD development and severity remain poorly understood (5–9).

AIEC drive intestinal inflammation and exacerbate colitis by adhering to and invading the intestinal epithelium of the host (10–14). Although no genetic markers have been identified to definitively differentiate AIEC from commensal *E. coli*, AIEC pathogenesis has been linked to virulence factors, such as fimbriae and pili, and to nutrient utilization and metabolism (15–19). Iron, for example, has been strongly correlated with AIEC virulence, and reducing the availability of fucose, an intestinal mucin glycan and bacterial carbon source, resulted in a significant attenuation of AIEC-mediated inflammation and colitis severity (15, 20). Host-derived ethanolamine and microbiota-derived propionate have both been associated with elevated intestinal inflammation in AIEC-colonized CD patients. Specifically, propionate has been linked to increased AIEC adhesion, invasion, and biofilm formation (21–24).

Propionate is generated from the metabolism of propanediol, a byproduct of fucose and rhamnose catalysis (25–27). Notably, the propanediol metabolic operon *pdu*, while not ubiquitous across all AIEC strains, appears more frequently in the AIEC accessory genome than in the genomes of commensal *E. coli* (15, 17, 28). The *pdu* operon canonically encodes a diol dehydratase and L-threonine kinase that together convert propanediol to propionate in a vitamin B_12_-dependent process; however, the enzymatic machinery is necessary but insufficient for propanediol metabolism (29, 30). Studies of *Salmonella enterica* Typhimurium demonstrated the importance of bacterial microcompartments in sequestering toxic aldehyde intermediates formed during propanediol metabolism, finding that *pdu*-associated microcompartments were visible in minimal media supplemented with propanediol, but not in minimal media supplemented with glucose (31). The *pduB* gene encodes two overlapping proteins, the full-length PduB and the truncated PduB′, which lacks PduB’s 37 N-terminal amino acids (32). Both proteins, collectively referred to as PduBB′, together represent the most abundant structural component of the molecular shell enclosing the Pdu propanediol dehydratase complex, and the N-terminus of PduB has been shown to physically link the microcompartment shell to the enzymatic propanediol dehydratase complex within (32, 33). Disruption solely of the PduB N-terminus culminated in the assembly of empty bacterial microcompartments lacking the requisite enzymatic machinery for propanediol metabolism (29, 33, 34). Deletion of *pduB* in its entirety likewise resulted in the formation of comparatively small, empty microcompartments, although an earlier study observed a near total abrogation of microcompartment formation alongside a significant growth defect when propanediol served as the sole carbon source (29, 34, 35). Furthermore, in media where propanediol served as the sole carbon source, *S. enterica* Typhimurium without *pduB* depleted propanediol more slowly than did the wild type (34). In AIEC, deletion of the propanediol dehydratase complex large subunit *pduC* significantly decreased AIEC-mediated intestinal inflammation and colitis severity in mice. Deletion of the propanediol microcompartment component *pduB*, as well as *pduC*, has been correlated with substantially reduced AIEC invasion as well (15, 36).

Although the relationship between propanediol metabolism and AIEC pathogenesis has been well established, the mechanism underlying this relationship remains largely unexplored. Vitamin B_12_ represents a promising candidate for mediating this relationship, as it is a key cofactor in propanediol metabolism. Furthermore, vitamin B_12_ deficiencies have been observed in patients with CD at a higher frequency than in healthy individuals (37, 38). These reduced levels of serum vitamin B_12_ in CD patients are often ascribed to inflammation, which prevents the absorption of various nutrients. However, gut bacteria have previously been correlated with variations in serum nutrient concentrations, suggesting that vitamin B_12_ levels may also fluctuate as a consequence of microbiota-associated modulation of nutrient availability (39). Diet supplementation with a cocktail of methyl donors, including vitamin B_12_, has also been associated with reduced AIEC colonization in humanized carcinoembryonic antigen bacterial artificial chromosome 10 (CEABAC10) mice, although the effects of vitamin B_12_ in isolation and in a CD mouse model have yet to be explored (40). These findings cumulatively suggest that AIEC may influence host vitamin B_12_ availability through microbe-associated modulation of nutrient availability, affecting AIEC propanediol metabolism and, by extension, AIEC pathogenesis.

Herein, we highlight the necessity of the propanediol metabolic microcompartment gene *pduB* for AIEC virulence and its intracellular life cycle. We show that deletion of *pduB* in the AIEC strain NRG857c significantly decreases bacterial invasion of Caco-2 intestinal epithelial cells without impacting bacterial growth or biofilm formation *in vitro*. We further demonstrate that AIEC-mediated disruption of barrier integrity and transepithelial electrical resistance in Caco-2 monolayers requires *pduB*. Extending these findings *in vivo*, we reveal that deletion of *pduB* reduces inflammation, as well as spontaneous and piroxicam-induced colitis in *Il10^−/^*^−^ mice. Finally, we uncover a relationship between vitamin B_12_ availability and AIEC virulence. We identify an inverse relationship between NRG857c propanediol metabolism and host serum levels of vitamin B_12_, as *Il10^−/^*^−^ mice colonized with NRG857c Δ*pduB* exhibit significantly more circulating vitamin B_12_ than mice colonized with wild-type NRG857c. Importantly, supplementation of vitamin B_12_ significantly inhibits AIEC invasion of intestinal epithelial cells and significantly attenuates inflammation and colitis severity in *Il10^−/^*^−^ mice colonized by both wild-type NRG857c and NRG857c Δ*pduB* strains. Taken together, these findings reveal how *pduB* and the propanediol metabolic microcompartment contribute to AIEC pathogenesis and host-microbe interactions involving essential micronutrients, such as vitamin B_12_, while highlighting the role of microbial and host-associated nutrient availability in AIEC carbon metabolism and pathogenesis.

## RESULTS

### *pduB* promotes AIEC invasion of intestinal epithelial cells

Because AIEC drive pathogenesis in IBD, particularly Crohn’s disease, by promoting chronic mucosal inflammation through adhesion to and invasion of the intestinal epithelium, we investigated the genetic factors underlying this invasive phenotype (5, 41–45). Previously, we constructed a comprehensive pangenome comprising 168 *E. coli* strains isolated from IBD patients (36). Comparative genomic analysis revealed a significant enrichment of genes involved in 1,2-propanediol metabolism among highly invasive clinical strains (36). Notably, these genes are clustered within a genomic island in the prototypical AIEC strain NRG857c, suggesting a potential role in host-pathogen interactions (36).

**Fig 1:**
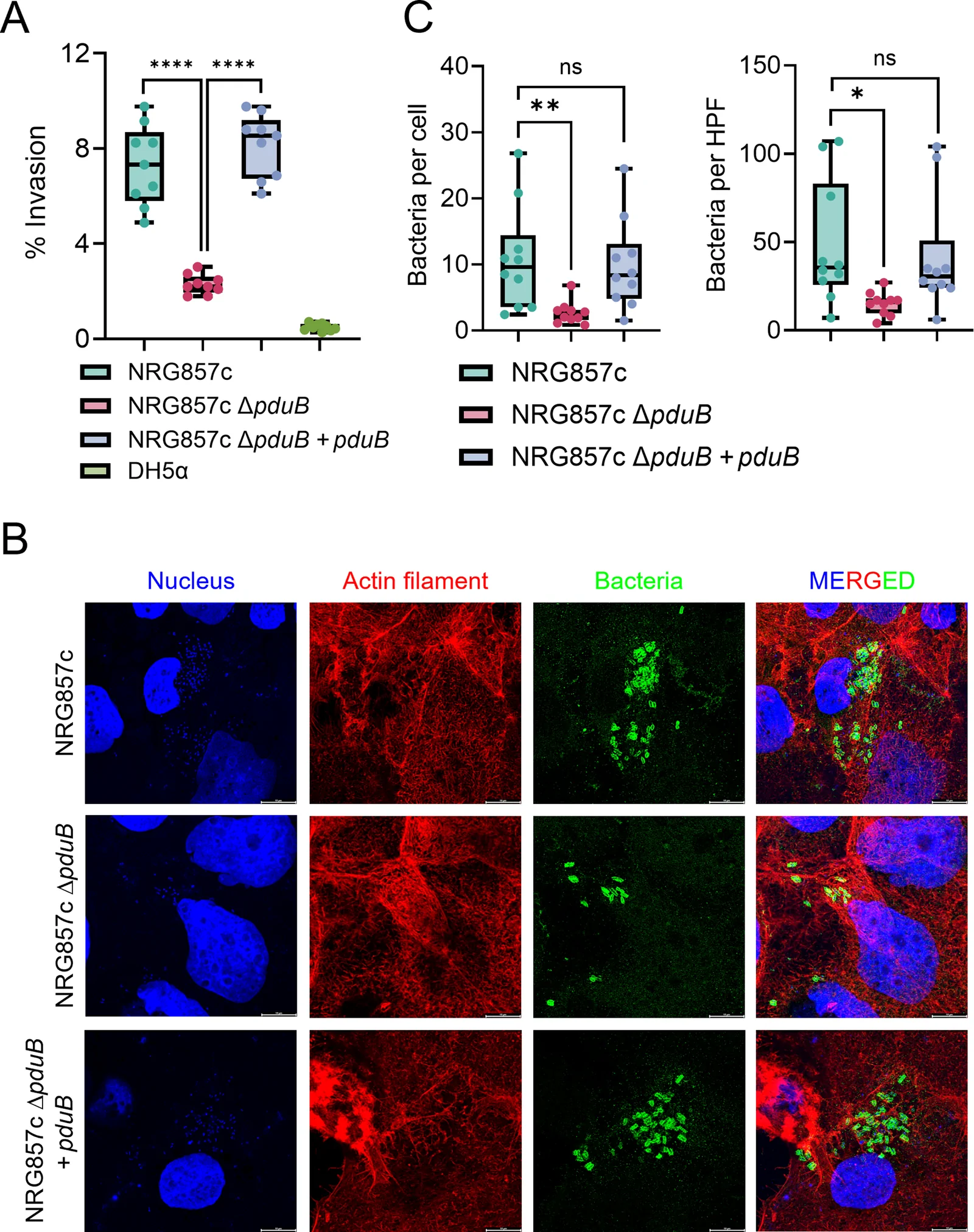
Deletion of *pduB* decreases invasion in intestinal cell line. (**A**) Caco-2 cells were seeded into collagen-coated 24-well plates. On day 3, cells were infected with AIEC strains NRG857c, NRG857c ∆*pduB*, NRG857c ∆*pduB + pduB,* and DH5α at a multiplicity of infection (MOI) = 10 for 3 h. Following amikacin treatment (240 μg/mL, 1 h), cells were lysed with 1% Triton X-100, and intracellular bacteria were quantified by serial dilution and plating on LB agar. (**B**) Caco-2 cells were seeded onto collagen-coated coverslips and infected with the same AIEC strains as in panel **A** under identical conditions. After amikacin treatment, cells were fixed, permeabilized, and immunostained with anti-*E*. *coli* LPS antibody, followed by Alexa Fluor 488-conjugated secondary antibody and rhodamine-phalloidin. Images were acquired using a Leica confocal microscope at 63× magnification. (**C**) Intracellular bacteria were quantified from at least 10 high-power fields (HPFs). Statistical significance for comparisons involving multiple groups was assessed by one-way ANOVA, and comparisons involving two groups were analyzed using an unpaired 2-tailed Student’s *t*-test.

Within this operon, *pduC* has previously been implicated in promoting Th17- and IL-1β-mediated intestinal inflammation (15). To further elucidate the function of this locus, we generated a targeted deletion of *pduB*, a core structural gene within the *pdu* operon (29, 34, 35, 46). Deletion of *pduB* in NRG857c (NRG857c Δ*pduB*) resulted in a marked reduction of epithelial cell invasion into Caco-2 cells (Fig. 1A). This invasion defect was restored by genetic complementation (NRG857c Δ*pduB + pduB*), confirming the functional importance of *pduB* in mediating host cell invasion (Fig. 1A). Consistent with these findings, confocal immunofluorescence microscopy demonstrated a substantial reduction in the number of intracellular bacteria in the NRG857c Δ*pduB* strain compared to the complement (+*pduB*) and wild-type strains (Fig. 1B and C). Of note, deletion of *pduB* did not affect bacterial growth or biofilm formation compared to the wild-type strain (Fig. S1A through C).

### *pduB* deficiency attenuates AIEC-induced colitis in *Il10^−/^*^−^ mice

**Fig 2:**
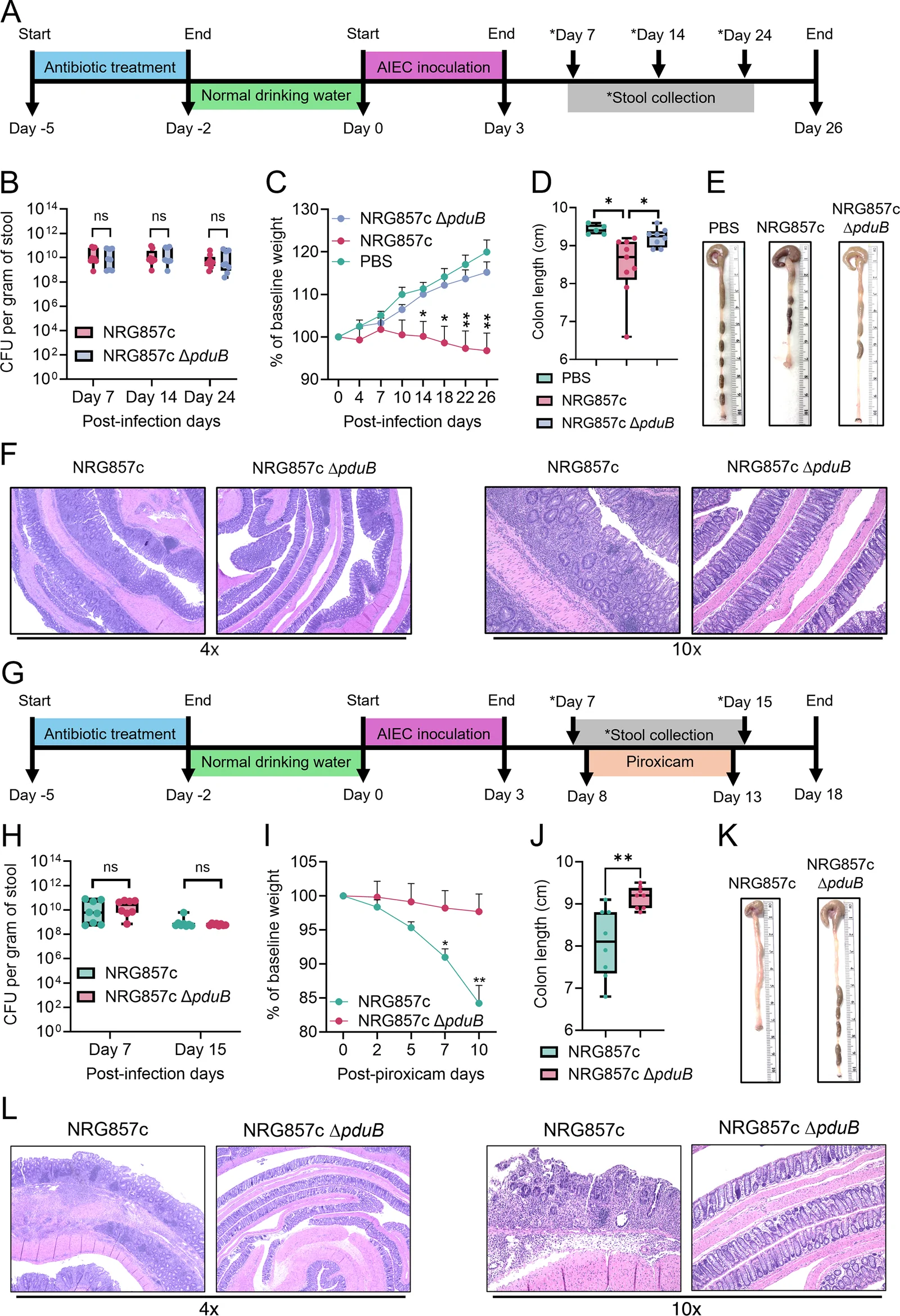
Deletion of *pduB* in AIEC strain NRG857c reduces spontaneous colitis in *Il10^−/^*^−^ mice. (**A**) Schematic outline of the study. (**B**) Antibiotic-treated mice were colonized with AIEC strains NRG857c and NRG857c ∆*pduB*. On days 7, 14, and 24 post-colonization, feces were collected, plated on LB agar, and colony-forming units (CFUs) were quantified. (**C**) Body weight changes were recorded on the indicated days. (**D–F**) On day 26, mice were sacrificed, and colon length was measured (**D and E**). Colon tissues were analyzed by hematoxylin and eosin (H&E) staining, and images were captured using a Keyence microscope at 4× and 10× magnification (**F**). (**G**) Schematic outline of the study. (**H**) Antibiotic-treated *Il10^−/^*^−^ mice were colonized with AIEC strains NRG857c and NRG857c ∆*pduB*. Stool samples were collected on days 7 and 15 post-colonization, plated on LB agar plates, and CFUs were quantified. (**I**) On day 8 post-colonization, *Il10^−/^*^−^ mice were fed with a piroxicam-fortified diet, and body weight was monitored. (**J–L**) On day 10 following piroxicam treatment, mice were sacrificed. Colon length was measured (**J and K**), and colon tissue sections were analyzed by H&E staining (**L**). Two-way ANOVA was used to assess comparisons involving multiple groups over time, and unpaired two-tailed Student’s *t*-test was used for endpoint comparisons. Comparisons involving only two groups were analyzed using an unpaired two-tailed Student’s *t*-test, as appropriate.

Our invasion assays demonstrated that the *pduB*-deficient NRG857c strain exhibited significantly reduced invasion of intestinal epithelial cells compared with the wild-type NRG857c strain (Fig. 1A through C). To evaluate whether *pduB’s* effect on invasion *in vitro* translated to an *in vivo* phenotype, IL-10 knockout (*Il10^−/−^*) mice were colonized with either wild-type NRG 857c, NRG857c Δ*pduB*, or PBS (uncolonized control) for 26 days after treatment with antibiotics to enhance engraftment (Fig. 2A). To monitor bacterial colonization, fecal samples were collected on days 7, 14, and 24 post-inoculation, and colony-forming units (CFUs) were measured by plating serial dilutions on selective LB agar plates (Fig. 2A). No significant difference in fecal CFU counts was observed between groups, indicating that *pduB* deletion does not impair bacterial growth or colonization capacity *in vivo* (Fig. 2B).

Despite similar colonization levels, *Il10^−/^*^−^ mice colonized with NRG857c exhibited more severe clinical and histological signs of colitis than those colonized with the Δ*pduB* mutant. NRG857c-colonized mice showed significantly decreased weights throughout the colonization period compared to Δ*pduB*-colonized mice (Fig. 2C). Notably, approximately 45% of the mice colonized with NRG857c developed rectal prolapse, a clinical sign of severe colitis, whereas this phenotype was absent in the Δ*pduB*-colonized group (Fig. S2A). In addition, colons from NRG857c-colonized mice were markedly shorter than those from Δ*pduB*-colonized mice (Fig. 2D and E). Histopathological examination of H&E-stained colon sections revealed extensive epithelial damage, crypt hyperplasia, and dense inflammatory cell infiltration in NRG857c-colonized mice (Fig. 2F). In contrast, Δ*pduB*-colonized mice displayed only mild to moderate inflammation with largely preserved epithelial architecture (Fig. 2F). Collectively, these findings demonstrate that *pduB* contributes to the ability of AIEC to induce intestinal inflammation and disease severity in *Il10^−/^*^−^ mice, independent of bacterial colonization efficiency.

In IBD, multiple signaling pathways, such as MAPK, NF-κB, PI3K/AKT, and JAK/STAT, drive chronic intestinal inflammation by mediating cytokine-driven immune activation, epithelial stress responses, macrophage polarization, and the transcription of pro-inflammatory genes that together sustain and amplify mucosal injury (47–49). To interrogate the underlying cause of reduced inflammation observed in *pduB*-deficient AIEC-colonized mice, we analyzed key cytokines involved in intestinal inflammation. The expression of pro-inflammatory cytokine genes *Il6*, *Il1b*, and *Tnf-a* was significantly higher in colonic tissues from mice colonized with wild-type NRG857c compared with those Δ*pduB*-colonized or PBS-treated controls (Fig. S2B). Interestingly, expression of *Ptgs2* (encoding COX-2) did not differ among the groups. This result may indicate that, although *Il6* is a potent pro-inflammatory cytokine capable of inducing COX-2 expression under certain contexts, in this model of AIEC-induced colitis, *Ptgs2* expression is either regulated independently of *Il6* signaling or requires additional stimuli beyond *Il6* activation. Together, these results demonstrate that *pduB* promotes the upregulation of key pro-inflammatory cytokines during AIEC-induced intestinal inflammation.

To validate our *in vivo* findings, we also used a piroxicam-induced *Il10^−/^*^−^ mouse model. The piroxicam-fortified diet contains a non-steroidal anti-inflammatory drug (NSAID) that induces colitis in *Il10^−/^*^−^ mice. This model mimics key immunopathological and barrier dysfunction features seen in human IBD, making it a robust tool for mechanistic and therapeutic studies of colitis (50–55). *Il10^−/^*^−^ mice were pre-treated with antibiotics and were colonized with either wild-type NRG857c or the Δ*pduB* mutant. Stable colonization was confirmed by culturing stool samples on antibiotic-selective LB agar plates at 7 days post-inoculation (Fig. 2G), and no difference was observed between the two groups (Fig. 2H), suggesting that *pduB* deficiency does not affect colonization ability *in vivo* when there is little to no inflammation. On day 8 post-colonization, mice were fed with piroxicam-fortified diet for 5 consecutive days to induce colitis (Fig. 2G), and body weight was monitored daily. Similar to day 7, no difference in colonization was observed at day 15 (Fig. 2H), suggesting that induction of inflammation did not affect colonization. However, mice colonized with wild-type NRG857c exhibited significantly greater weight loss compared with those colonized with the Δ*pduB* mutant (Fig. 2I). Colon length was significantly longer in Δ*pduB*-colonized mice than in wild-type NRG857c-colonized *Il10^−/^*^−^ mice (Fig. 2J and K), suggesting reduced disease burden. Histopathological analysis of H&E-stained colon sections further supported these findings, revealing severe epithelial damage, crypt distortion, and inflammatory cell infiltration in colons from NRG857c-colonized mice (Fig. 2L). In contrast, Δ*pduB*-colonized mice displayed substantially reduced epithelial injury and inflammatory changes (Fig. 2L). Together, these results demonstrate that *pduB* contributes to AIEC-induced exacerbation of piroxicam-mediated colitis in *Il10^−/^*^−^ mice and that its deletion mitigates disease severity independent of bacterial colonization.

### *pduB* contributes to AIEC-induced disruption of intestinal barrier function

**Fig 3:**
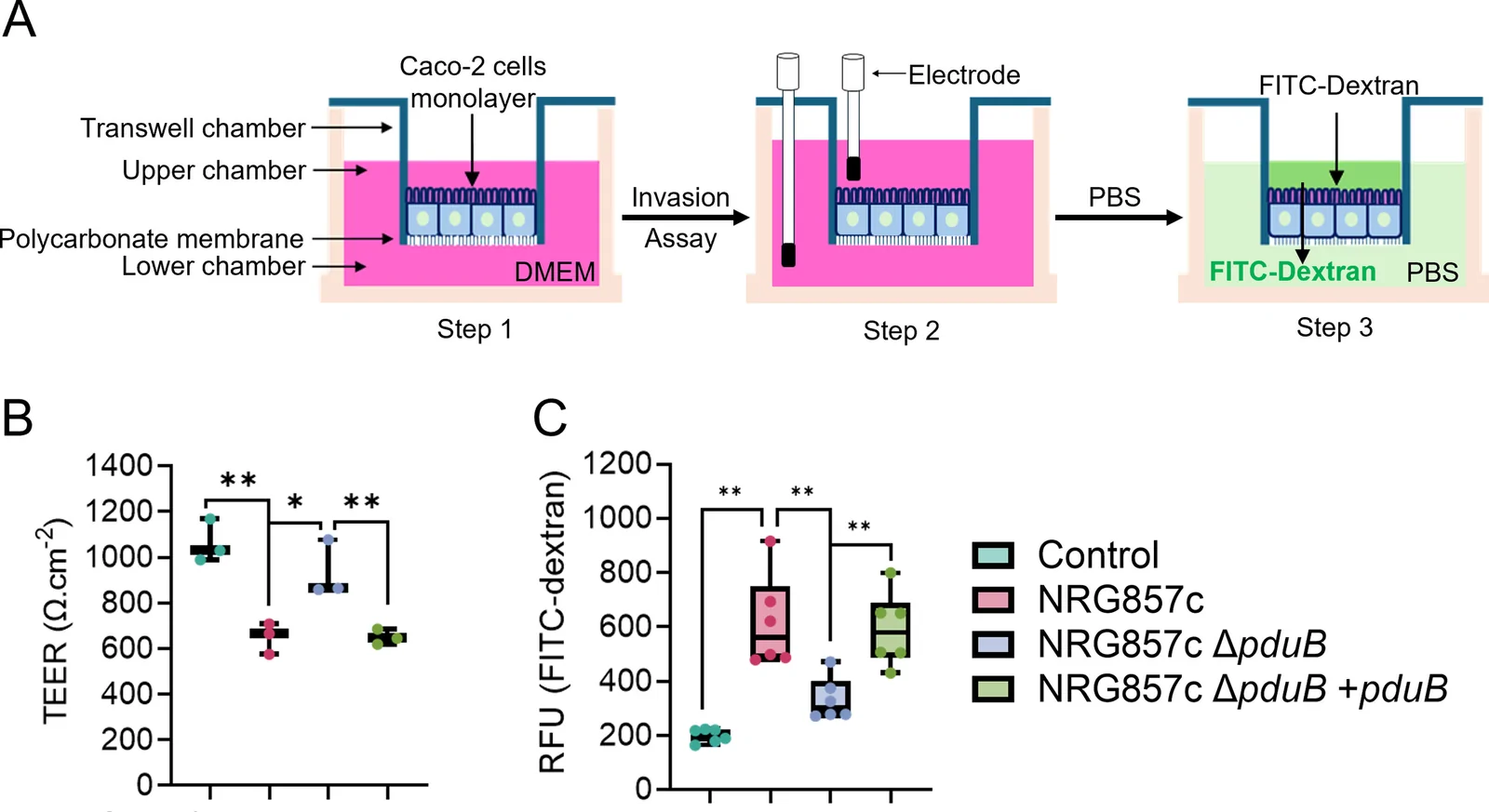
Loss of *pduB* reduces AIEC effects on epithelial resistance and barrier integrity in intestinal cells. (**A**) Experimental schematic. (**B and C**) Caco-2 cells were seeded into collagen-coated 24-well transwell plates. On day 5 (or upon reaching >90% confluence), cells were infected with NRG857c, NRG857c ∆*pduB*, and NRG857c ∆*pduB + pduB* at an MOI of 10 for 3 h. Following amikacin treatment (240 μg/mL, 1 h), transepithelial electrical resistance (TEER) was measured using a Millicell ERS-2 voltohmmeter, as shown in step 2 (**B**). Cells were subsequently washed with PBS, and FITC-dextran permeability was assessed by adding FITC-dextran (1 mg/mL; 100 μg/well) to the upper chamber. Following a 20-min incubation at 37°C, fluorescence in the lower chamber was measured using a plate reader (Gen5) (**C**). One-way ANOVA was used to assess comparisons involving multiple groups at single time points. Comparisons involving only two groups were analyzed using an unpaired 2-tailed Student’s *t*-test.

To assess the impact of *pduB* on intestinal epithelial barrier integrity, we performed transepithelial electrical resistance (TEER) and FITC-dextran permeability assays (Fig. 3A). TEER, a widely used quantitative method to monitor tight junction function in Caco-2 monolayers, provides a dynamic measure of barrier strength over time (56–58). FITC-dextran flux evaluates paracellular leakage, reflecting size-selective barrier disruption (56–58). TEER measurements revealed significantly lower resistance in Caco-2 cells infected with wild-type NRG857c or the complemented NRG857c Δ*pduB + pduB* strain compared with cells infected with the NRG857c Δ*pduB* mutant (Fig. 3B), suggesting that the presence of *pduB* compromises barrier function. Consistently, FITC-dextran flux across the epithelium was significantly higher in the wild-type and *pdu*-complemented groups compared with the Δ*pduB* mutant (Fig. 3C), further confirming increased epithelial permeability. These results demonstrate that *pduB* contributes to AIEC-mediated disruption of the intestinal barrier, whereas its deletion attenuates bacterial ability to compromise epithelial integrity.

### Vitamin B_12_ suppresses *pdu*-dependent AIEC invasion and barrier dysfunction

**Fig 4:**
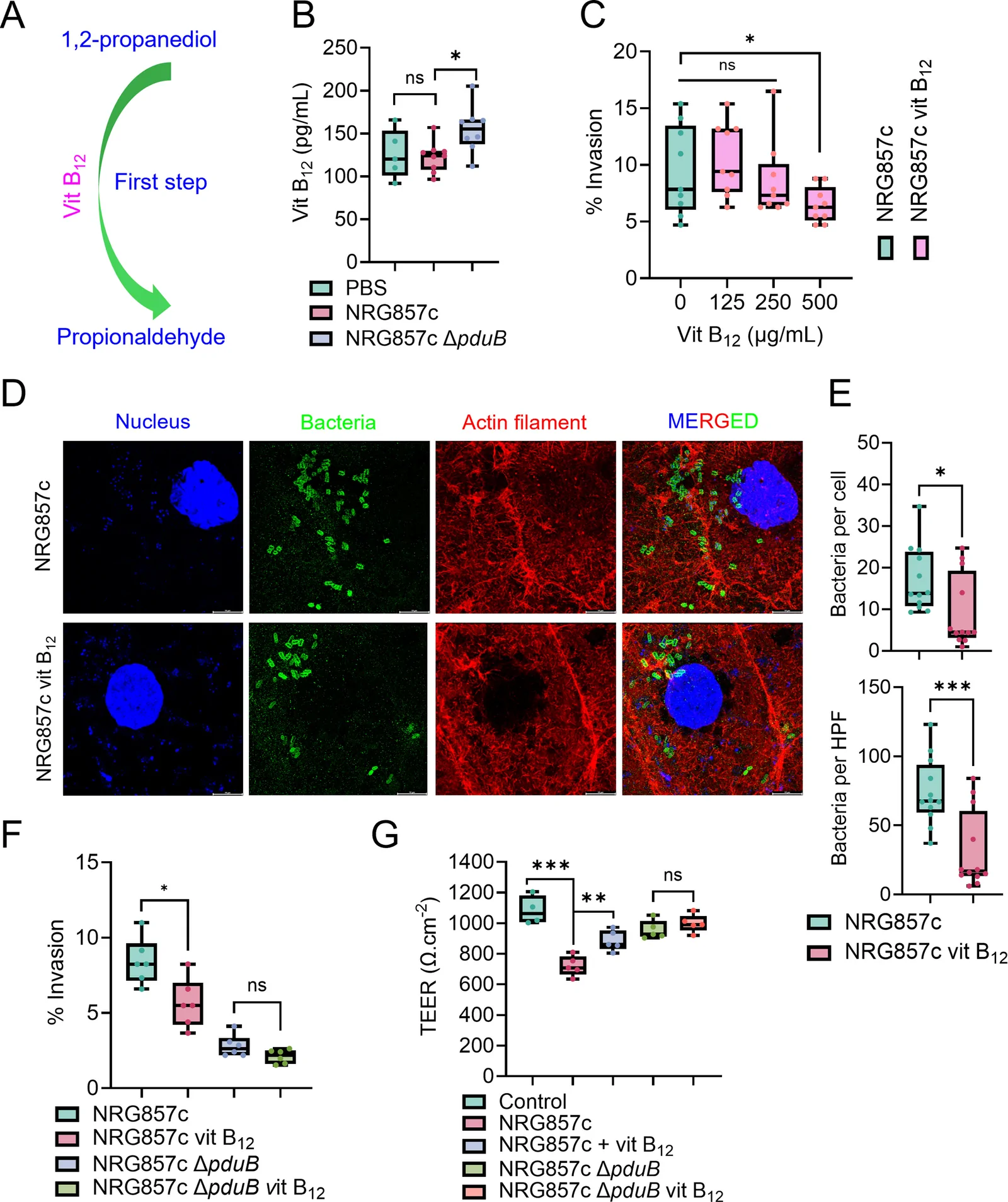
Vitamin B_12_ inhibits NRG857c invasion and barrier dysfunction in a *pdu*-dependent manner. (**A**) Role of vitamin B_12_ in 1,2-propanediol degradation. (**B**) Antibiotic-treated mice were colonized with AIEC strains NRG857c and NRG857c ∆*pduB*. On day 26, mice were euthanized, blood was collected for serum separation, and serum vitamin B_12_ concentrations were measured using an ELISA kit. (**C**) Caco-2 cells were seeded into collagen-coated 24-well plates. On day 3, cells were replenished with antibiotic-free medium containing vitamin B_12_ (0, 125, 250, or 500 µg/mL) and infected with NRG857c (MOI = 10) for 3 h. Following amikacin treatment (240 µg/mL, 1 h), cells were lysed with 1% Triton X-100, and intracellular bacteria were quantified by serial dilution and plating on LB agar. (**D and E**) Caco-2 cells grown on collagen-coated coverslips were infected with NRG857c in the presence or absence of vitamin B_12_ (500 µg/mL) as described in **panel C**. Following amikacin treatment, cells were fixed, immunostained for *E. coli* LPS, counterstained with rhodamine-phalloidin and DAPI, and imaged by confocal microscopy (**D**). Intracellular bacteria were quantified from at least 12 high-power fields (HPFs) (**E**). (**F**) Caco-2 cells were infected with wild-type NRG857c or the ∆*pduB* mutant in the presence or absence of vitamin B_12_ (500 µg/mL), and bacterial invasion was quantified using the amikacin protection assay as described in **panel C**. (**G**) Barrier function was assessed in Caco-2 transwell monolayers infected with NRG857c or the ∆*pduB* mutant in the presence or absence of vitamin B_12_ (500 µg/mL). Following amikacin treatment, transepithelial electrical resistance (TEER) was measured using a Millicell ERS-2 voltohmmeter. One-way ANOVA was used to assess comparisons involving multiple groups at single time points. Unpaired two-tailed Student's *t*-test was used for endpoint comparisons and for comparisons involving only two groups, as appropriate.

Our results demonstrated that deletion of *pduB*, a core structural component of the bacterial microcompartment, plays a critical role in regulating the invasive capacity of NRG857c in Caco-2 cells (29, 34, 35, 46). Given that vitamin B_12_ (cobalamin) is the essential cofactor for the B_12_-dependent diol dehydratase that converts 1,2-propanediol to propionaldehyde (Fig. 4A), the key metabolic reaction encoded by the *pdu* operon, we next sought to determine whether vitamin B_12_ influences AIEC-induced colitis in *Il10^−/^*^−^ mice (31, 59). To test this hypothesis, vitamin B_12_ levels were measured in *Il10^−/^*^−^ mice colonized with wild-type NRG857c, the Δ*pduB* mutant, or PBS as a control. We found that vitamin B_12_ concentrations were significantly higher in the blood serum of mice colonized with the Δ*pduB* mutant compared with those colonized with NRG857c but not compared with PBS controls (Fig. 4B). These findings suggest that disruption of the *pdu* pathway may reduce bacterial utilization of vitamin B_12_ during colonization, thereby increasing systemic vitamin B_12_ availability to the host.

Since we observed elevated vitamin B_12_ levels in the blood serum of Δ*pduB*-colonized mice, we wanted to determine whether vitamin B_12_ directly affects AIEC invasion. We performed invasion assays using Caco-2 cells in the presence of vitamin B_12_ at concentrations of 125, 250, and 500 μg/mL. These concentrations were selected to represent moderate and high supra-physiological exposure levels based on prior *in vitro* studies showing that Caco-2 cells maintain >90% viability at elevated vitamin B_12_ concentrations (500 µg/mL) (60, 61). Notably, only the high dose of vitamin B_12_ (500 μg/mL) significantly reduced the invasion efficiency of wild-type NRG857c compared with untreated controls (Fig. 4C), indicating that lower experimental levels of vitamin B_12_ are insufficient to impact bacterial invasion. Confocal microscopy also revealed a significantly reduced number of intracellular bacteria in Caco-2 cells treated with 500 μg/mL vitamin B_12_ relative to untreated cells (Fig. 4D and E). Together, these findings indicate that vitamin B_12_ can negatively regulate AIEC invasion of intestinal epithelial cells, but only at higher concentrations. These results further suggest that vitamin B_12_ supplementation at higher doses may render AIEC bacteria less invasive. To determine whether the anti-virulence effects of vitamin B_12_ require an intact *pdu* pathway, we next examined the impact of vitamin B_12_ on invasion and epithelial barrier function using both wild-type NRG857c and the Δ*pduB* mutant.

To determine whether the inhibitory effect of vitamin B_12_ on AIEC invasion requires *pduB*, we performed additional invasion assays using wild-type NRG857c and the Δ*pduB* mutant in the presence or absence of vitamin B_12_ (500 μg/mL). Consistent with our initial observations, vitamin B_12_ supplementation significantly reduced invasion of wild-type NRG857c into Caco-2 cells (Fig. 4F). In contrast, vitamin B_12_ treatment did not further reduce invasion of the Δ*pduB* mutant, and no significant difference was observed between Δ*pduB* and Δ*pduB* treated with vitamin B_12_ (Fig. 4F). These findings suggest that the suppressive effect of vitamin B_12_ on AIEC invasion is largely dependent on a functional *pdu* pathway and further support the conclusion that vitamin B_12_ modulates *pdu*-mediated virulence-associated functions.

To determine whether the protective effect of vitamin B_12_ on epithelial barrier integrity is dependent on *pduB*, we performed additional TEER assays using Caco-2 monolayers infected with wild-type NRG857c or the Δ*pduB* mutant in the presence or absence of vitamin B_12_ supplementation (500 μg/mL). Consistent with our previous findings, infection with wild-type NRG857c significantly reduced TEER compared with uninfected control monolayers (Fig. 4G). Notably, vitamin B_12_ supplementation significantly attenuated the reduction in TEER caused by wild-type NRG857c (Fig. 4G). In contrast, vitamin B_12_ treatment did not further improve TEER in monolayers infected with the Δ*pduB* mutant, as TEER values remained comparable between Δ*pduB*-infected cells and Δ*pduB*-infected cells treated with vitamin B_12_ (Fig. 4G). Together, these findings suggest that the protective effects of vitamin B_12_ on AIEC virulence and epithelial barrier dysfunction are largely dependent on an intact *pdu* pathway and further support a mechanistic link between vitamin B_12_ availability and *pdu*-mediated pathogenicity.

### Vitamin B_12_ attenuates AIEC-induced colitis in *Il10^−/^*^−^ mice

**Fig 5:**
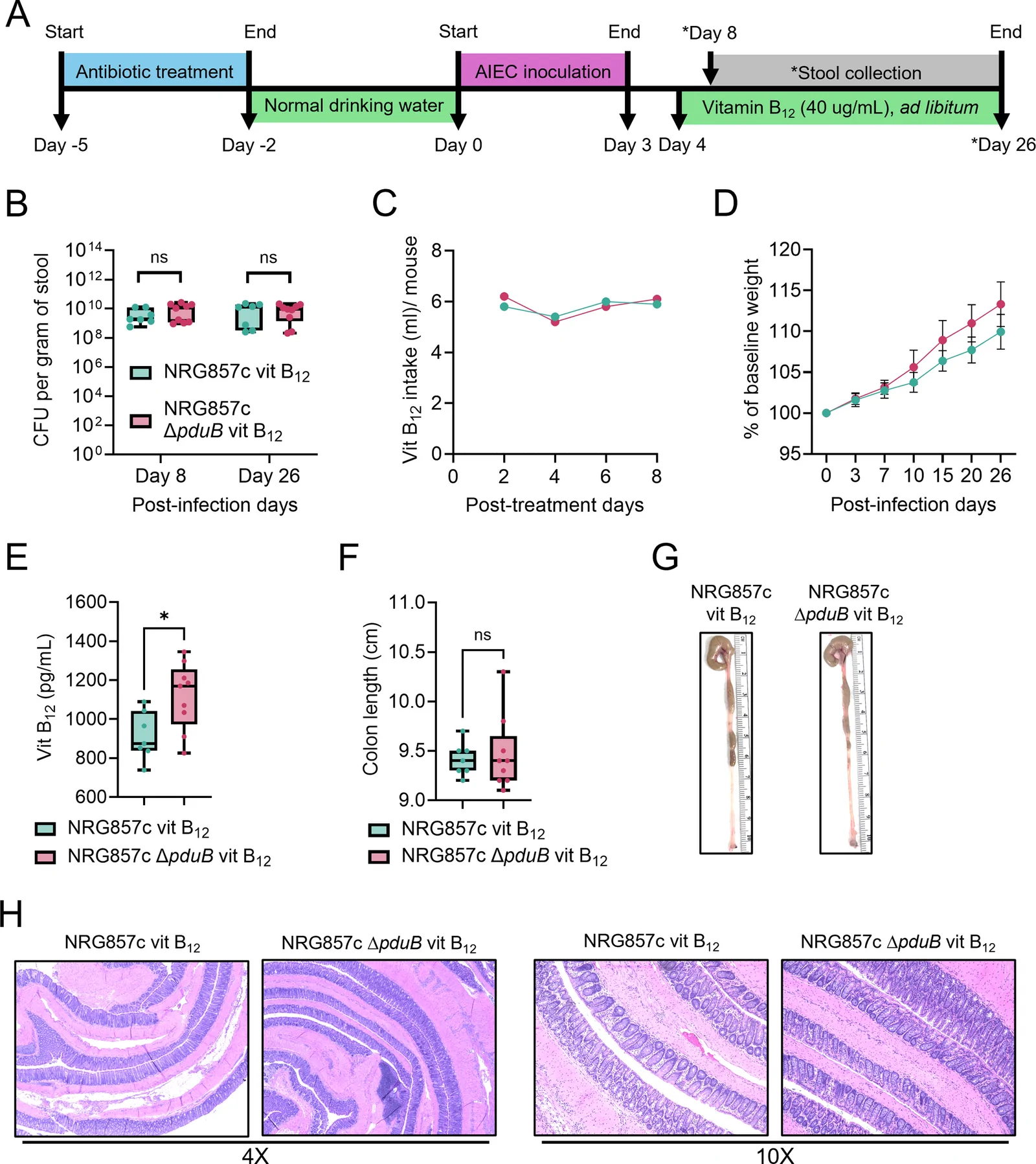
Vitamin B_12_ supplementation reduces NRG857c-induced colitis in *Il10^−/^*^−^ mice. (**A**) Schematic outline of the study. (**B**) Antibiotic-treated mice were colonized with NRG857c and NRG857c ∆*pduB*. Feces were collected on days 8 and 26 post-colonization, plated on LB agar, and CFUs were quantified. (**C**) Starting on day 4 post-colonization, mice were provided with vitamin B_12_ in their drinking water (*ad libitum*). (**D**) Body weight changes were recorded throughout the experiment. (**E–H**) On day 26 post-colonization, mice were sacrificed. Serum vitamin B_12_ levels were measured using an ELISA kit (**E**). Colon length was measured (**F and G**). Colon tissues were analyzed histologically following H&E staining, and images were captured at 4× and 10× magnification using a Keyence microscope (**H**). Two-way ANOVA was used to assess comparisons involving multiple groups over time, with appropriate multiple comparisons testing. Unpaired two-tailed Student’s *t*-test was used for endpoint comparisons, as appropriate.

To further investigate the role of vitamin B_12_ in AIEC-induced colitis, we conducted *in vivo* experiments in antibiotic-treated *Il10^−/^*^−^ mice colonized with either wild-type NRG857c or the Δ*pduB* mutant (Fig. 5A). Bacterial colonization was confirmed by plating stool samples collected at days 8 and 26 post-colonization on LB agar plates (Fig. 5A), which revealed no significant difference in fecal bacterial CFU counts between groups (Fig. 5B). Starting on day 4 post-colonization, both groups received vitamin B_12_ (40 μg/mL) in their drinking water *ad libitum* until the end of the experiment (Fig. 5A). This dose was selected based on a prior study in which a 40 μg/mL dose in mice was used to approximate the human-equivalent dose of vitamin B_12_ supplementation (62). To ensure that both groups received equal amounts of vitamin B_12_, water intake was monitored daily until day 8 following vitamin B_12_ supplementation. Both wild-type and Δ*pduB*-colonized mice consumed comparable volumes of water, confirming equivalent vitamin B_12_ intake (Fig. 5C ; Fig. S3A).

Throughout the study period, including during vitamin B_12_ administration, body weights remained comparable between mice colonized with wild-type NRG857c and the Δ*pduB* mutant (Fig. 5D). Notably, serum vitamin B_12_ concentrations remained significantly higher in Δ*pduB*-colonized mice than in mice colonized with wild-type NRG857c despite vitamin B_12_ supplementation (Fig. 5E). Interestingly, mice colonized with NRG857c that received vitamin B_12_ supplementation showed no signs of rectal prolapse (Fig. S3B), and no significant differences in colon length were observed between the groups (Fig. 5F and G). Histopathological analysis of H&E-stained colon sections further confirmed normal intestinal architecture with no evidence of epithelial injury or inflammatory cell infiltration in either group (Fig. 5H). Together, these findings demonstrate that vitamin B_12_ supplementation, at the tested concentration, elevates serum vitamin B_12_ levels and attenuates AIEC-induced colitis in *Il10^−/^*^−^ mice. Collectively, these findings suggest that increased systemic availability of vitamin B_12_ can mitigate the pathogenic potential of AIEC and reduce disease severity in *Il10^−/^*^−^ mice.

### *pduB* promotes mucosal association during intestinal inflammation

**Fig 6:**
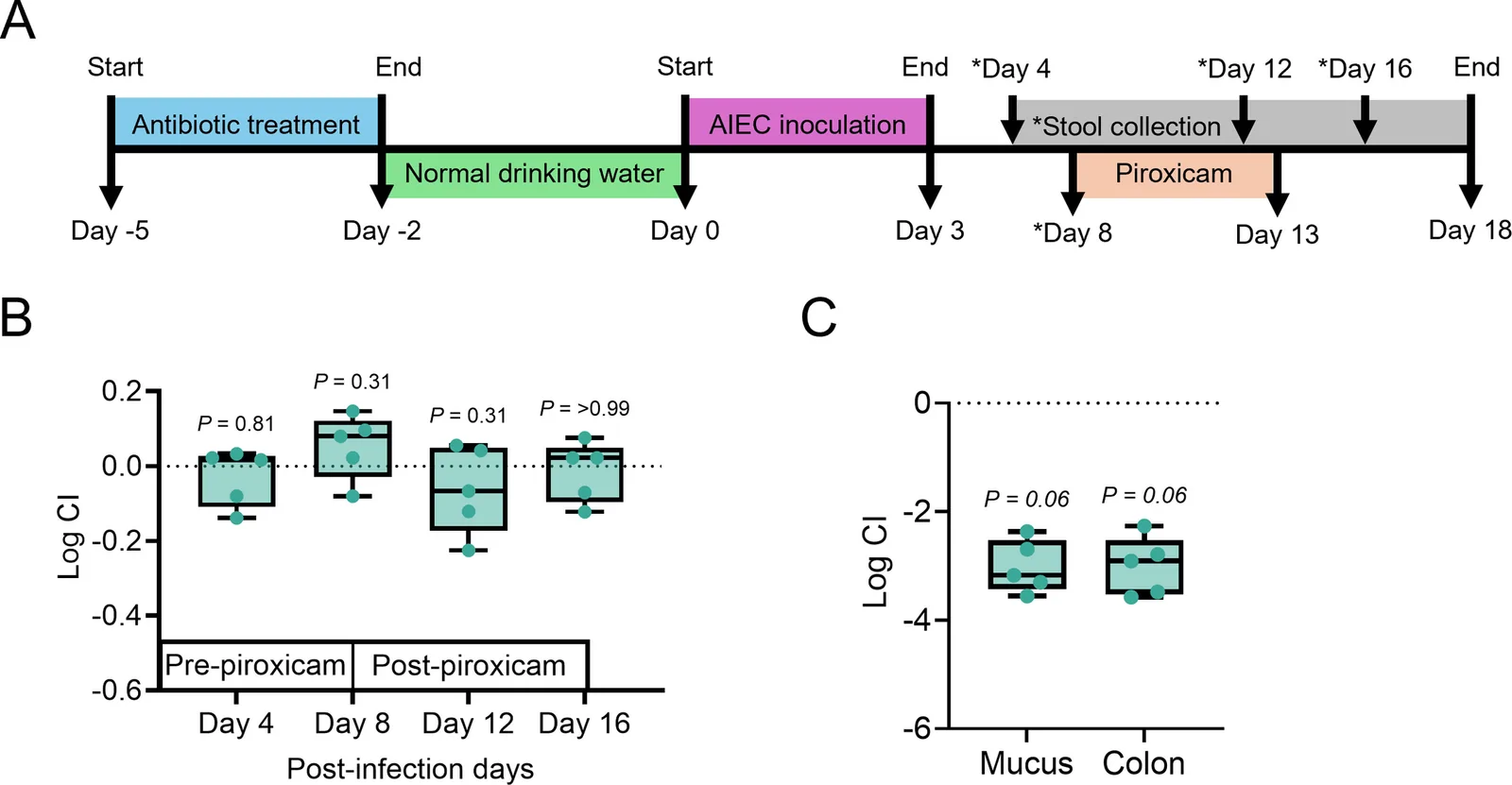
*pduB* promotes NRG857c mucosal association during intestinal inflammation. (**A**) Schematic outline of the study. Antibiotic-treated *Il10^−/^*^−^ mice were co-colonized with a 1:1 mixture of wild-type NRG857c and the isogenic Δ*pduB* mutant (1 × 10^8^ CFU of each strain per mouse) by oral gavage once daily for 3 consecutive days. (**B**) Fecal samples were collected on days 4, 8, 12, and 16 to assess luminal bacterial competition using log of the competitive index (log CI). Mice were switched to a piroxicam-fortified diet on day 8 to induce intestinal inflammation and were euthanized on day 18 (**A**). The colonic mucus layer was collected by gentle scraping, and colonic tissues were processed separately for bacterial quantification. (**C**) Bacterial competition in colonic mucus and colonic tissue samples was quantified as log (CI), representing the relative abundance of wild-type NRG857c relative to the Δ*pduB* mutant within each compartment. Wilcoxon signed-rank tests were performed to compare log CI values at each fecal sampling time point. For the colonic mucus and tissue analyses, the *P* value of 0.0625 represents the smallest attainable *P* value for a Wilcoxon signed-rank test with 5 mice.

As luminal bacterial counts were comparable between wild-type NRG857c and the Δ*pduB* mutant throughout our infection studies, we next sought to determine whether *pduB* specifically affects mucosal colonization during intestinal inflammation. *Il10^−/^*^−^ mice were co-colonized with wild-type NRG857c and the Δ*pduB* mutant before colitis induction via administration of piroxicam on day 8 (Fig. 6A). Fecal samples collected on days 4, 8, 12, and 16 post-colonization revealed no significant differences in luminal bacterial abundance between the two strains either prior to or after induction of colitis (Fig. 6B). However, although luminal colonization remained comparable throughout the experiment (Fig. 6B), wild-type NRG857c demonstrated a 3-log competitive advantage over the Δ*pduB* mutant in both the colonic mucus layer and colonic tissue (Fig. 6C). These findings suggest that *pduB* is not necessary for luminal colonization but promotes mucosal association and invasion during colitis.

To ensure that the observed vitamin B_12_-mediated protection in AIEC-colonized mice was not due to a general anti-inflammatory effect, we also supplemented AIEC-free piroxicam-treated *Il10^−/^*^−^ mice with vitamin B_12_. *Il10^−/^*^−^ mice were provided vitamin B_12_ supplementation in drinking water throughout the experimental period and were compared with untreated controls (Fig. S4A). Piroxicam administration induced significant weight loss indicative of active disease, and vitamin B_12_ supplementation did not significantly reduce weight loss during disease progression (Fig. S4B). Furthermore, no significant differences in colon length were observed between vitamin B_12_-treated and untreated mice (Fig. S4C and D). These findings suggest that vitamin B_12_ supplementation is insufficient to attenuate piroxicam-induced colitis and that the protective effects of vitamin B_12_ observed in our study are linked to modulation of AIEC-associated pathogenesis rather than generalized suppression of intestinal inflammation.

To determine whether intestinal inflammation alone affects systemic vitamin B_12_ levels, serum vitamin B_12_ concentrations were measured in control and piroxicam-treated *Il10^−/^*^−^ mice in the absence of AIEC colonization. Despite the development of colitis, as indicated by significant weight loss in piroxicam-treated mice compared with untreated controls (Fig. S4E), serum vitamin B_12_ concentrations were comparable between the two groups (Fig. S4F). These findings suggest that intestinal inflammation does not substantially alter circulating vitamin B_12_ levels in this model and support the interpretation that the elevated serum vitamin B_12_ levels observed in Δ*pduB*-colonized mice are associated with disruption of the *pdu* pathway rather than inflammation *per se*.

## DISCUSSION

The findings presented here collectively reveal a central role for the *pdu* operon and vitamin B_12_ in driving multiple pathogenic properties of AIEC relevant to Crohn’s disease. Previous comparative genomics of IBD-associated *E. coli* strains identified a strong enrichment of 1,2-propanediol metabolic genes among highly invasive AIEC isolates, implicating this locus as a determinant important for host cell invasion (36). The current work experimentally validates this association by demonstrating that *pduB*, a core structural gene required for constructing the bacterial microcompartment that hosts 1,2-propanediol metabolism (29, 34, 35, 46), is essential for AIEC invasion of intestinal epithelial cells. Deletion of *pduB* markedly reduced invasion *in vitro*, a defect fully restored by complementation, while bacterial growth, biofilm formation, and *in vivo* colonization remained unaffected. These observations suggest that *pduB* acts as a virulence determinant rather than a fitness gene in the lumen of the GI tract and contributes to AIEC penetration of host epithelial surfaces. Importantly, *pduB* was selected for targeted deletion because it encodes a major structural shell protein of the *pdu* bacterial microcompartment (29, 34, 35, 46). Previous studies have shown that loss of *pduB* disrupts microcompartment assembly and functionality, resulting in broad impairment of 1,2-propanediol utilization rather than affecting a single catalytic reaction (29, 34, 35).

The functional importance of *pduB* extends beyond invasion, as demonstrated in two complementary *Il10^−/^*^−^ mouse models. Despite equivalent luminal colonization, mice inoculated with the Δ*pduB* mutant developed significantly less intestinal inflammation compared with wild-type AIEC-colonized mice. Wild-type NRG857c triggered severe phenotypes, including body weight loss, rectal prolapse, colon shortening, and extensive epithelial and inflammatory damage, while Δ*pduB*-colonized animals exhibited only mild to moderate pathology. Similar findings in the piroxicam-induced colitis model reinforce the conclusion that *pduB* enhances AIEC-driven inflammation independently of its impact on colonization. Importantly, our *in vivo* competition experiments further demonstrate that *pduB* contributes specifically to mucosal association during intestinal inflammation, as luminal (fecal) colonization remained comparable between wild-type NRG857c and Δ*pduB* strains, whereas wild-type NRG857c exhibits a 3-log competitive advantage over the Δ*pduB* mutant within the colonic mucus layer and colonic tissue. These results suggest that the *pdu* microcompartment contributes directly to host-pathogen interactions that amplify inflammatory responses. In line with the reduced disease severity, Δ*pduB*-colonized mice showed lower expression of *Il6*, *Il1b*, and *Tnf-a*. Given that AIEC invasion is a known trigger of inflammatory signaling and cytokine production, the impaired invasion capacity of the Δ*pduB* strain is an attractive explanation for its attenuated inflammatory phenotype (44, 63–65). Interestingly, *Ptgs2* expression did not differ between groups, suggesting that the *pdu* operon does not broadly regulate inflammatory gene programs but rather specifically modulates select inflammatory circuits.

Epithelial barrier function, another hallmark of AIEC-associated pathology, was also influenced by *pduB* (63, 66, 67). Wild-type NRG857c induced significant barrier dysfunction *in vitro*, as indicated by reduced TEER and increased FITC-dextran permeability, whereas the Δ*pduB* mutant caused minimal disruption. This impaired ability to compromise the epithelial barrier is consistent with reduced invasion and inflammatory signaling, highlighting a coordinated role for *pduB* in modulating AIEC activities that collectively weaken mucosal defenses.

A novel and particularly intriguing aspect of this work is the discovery of an inverse relationship between *pduB*, vitamin B_12_ metabolism, and AIEC virulence. Because vitamin B_12_ serves as a cofactor for metabolic reactions within the *pdu* microcompartment, deletion of *pduB* may disrupt bacterial utilization of vitamin B_12_. Δ*pduB*-colonized mice showed substantially elevated circulating vitamin B_12_ levels, suggesting reduced microbial consumption or altered host-microbiota nutrient utilization. Functionally, exogenous vitamin B_12_ significantly inhibited AIEC invasion of epithelial cells, an effect confirmed by both amikacin protection assays and confocal microscopy. Notably, this inhibitory effect was observed only in wild-type bacteria, whereas Δ*pduB* strains were not further affected, indicating that the anti-invasive effect of vitamin B_12_ is dependent on an intact *pdu* pathway. These findings identify vitamin B_12_ as a regulator of AIEC invasion, likely by constraining metabolic activity linked to the *pdu* operon or altering bacterial physiology in a manner that dampens virulence. Vitamin B_12_ supplementation provided strong protection against AIEC-induced colitis in *Il10^−/^*^−^ mice, preventing weight loss, rectal prolapse, and tissue damage. Importantly, supplementation did not alter luminal colonization, indicating that its protective effect stems from suppression of bacterial virulence rather than pathogen burden, at least in the lumen. These results highlight vitamin B_12_ availability as a host-modifiable factor capable of attenuating AIEC pathogenicity and limiting intestinal inflammation. We further found that vitamin B_12_ supplementation did not significantly attenuate piroxicam-induced colitis in the absence of AIEC, supporting a pathogen-specific effect rather than a generalized anti-inflammatory role.

This study has several limitations that should be considered when interpreting the findings. While our data support a role for *pduB* in AIEC host interactions and disease modulation, the mechanistic links between propanediol metabolism, vitamin B_12_ availability, and host inflammatory responses were not fully dissected. In particular, the relative contribution of *pduB*-dependent microcompartment activity to epithelial barrier disruption and immune activation remains to be defined at the molecular level. Additionally, although we observed altered systemic vitamin B_12_ levels, we cannot definitively conclude direct host-microbe competition for vitamin B_12_ without direct uptake flux measurements. Our experimental models may not fully recapitulate the complexity and heterogeneity of the human gut environment, including microbial community interactions and dietary variability that influence vitamin B_12_ dynamics. Furthermore, in our *in vitro* experiments, we expect that the source of 1,2-propanediol is conversion from cell-secreted mucus via fermentation, but this was not confirmed experimentally. Finally, although vitamin B_12_ supplementation mitigated AIEC-driven pathology in our models, the translatability of this intervention to AIEC-positive IBD patients requires validation in clinically relevant settings. Future studies integrating multi-omics approaches and human-based models will be essential to address these limitations and refine the therapeutic potential of targeting bacterial metabolic pathways.

In summary, *pduB* may contribute to AIEC virulence not only via its role in propanediol metabolism, a pathway linked to bacterial invasion and inflammatory potential in the gut environment, but also through its use of vitamin B_12_ as a cofactor. Our findings suggest that *pduB* may impact AIEC-host interactions, including mucosal association, epithelial barrier integrity, immune activation, and B_12_-associated metabolic activity, with potential consequences for host vitamin B_12_ dynamics. These findings underscore the broader concept that bacterial metabolic pathways, particularly those involving microcompartment-dependent reactions, play a critical role in modulating host-pathogen interactions. They also provide a foundation for future research on how AIEC-host resource interactions influence AIEC pathogenesis and colonization. Moreover, the discovery that vitamin B_12_ supplementation can counteract AIEC-driven disease raises the possibility that metabolic interventions may offer therapeutic benefit in AIEC-positive IBD patients.

## MATERIALS AND METHODS

### Sex as a biological variable

Both male and female *Il10^−/^*^−^ mice (C57BL/6 background) were included in this study. Animals were age- and sex-matched across experimental groups to account for sex as a biological variable.

### Deletion and complementation of *pduB* in AIEC prototype strain NRG857c

A mutant of the adherent-invasive *Escherichia coli* (AIEC) strain NRG857c lacking the *pduB* gene was constructed using the Lambda Red recombineering method as previously described (68). In brief, electrocompetent NRG857c cells were first transformed with plasmid pTKRED (Addgene #41062) via electroporation and cultured aerobically on Luria-Bertani (LB) agar plates (Fisher) supplemented with spectinomycin (Goldbio) for selection. Subsequently, NRG857C/pTKRED were transformed with a PCR product generated using primers containing 40–50 bp of sequence homology (Table S1), using pKD13 as the DNA template. Recombinants were selected on LB plates containing 50 µg/mL kanamycin (Sigma), and *pduB* gene deletion was confirmed by PCR analysis and whole-genome sequencing. An electrocompetent *pduB* mutant of NRG857c was then transformed with pBAD24 carrying the full-length *pdu* operon and incubated at 37°C for 3 h to allow recovery and expression of antibiotic resistance. Cells containing pBAD24 were selected on LB agar plates supplemented with 100 µg/mL ampicillin. Successful complementation of the *pdu* operon was confirmed by PCR analysis of colonies grown on the ampicillin-containing LB plates.

### Bacterial invasion assay

We have previously described this protocol in detail (36). In brief, approximately 1 × 10^5^ Caco-2 cells were seeded into collagen-coated 24-well plates. On day 3 (or when cells reached >90% confluency), the monolayers were washed with warm, antibiotic, and serum-free DMEM. Cells were then infected with *E. coli* strains DH5α (negative control), NRG857c, NRG857c Δ*pduB*, and NRG857c Δ*pduB + pduB* using an MOI of 10. Following centrifugation at 500 × *g* for 15 min, the cells were incubated at 37°C in a 5% CO_2_ incubator for 3 h. After incubation, the cells were washed and incubated with serum-free DMEM containing 240 μg/mL amikacin for 1 h at 37°C in a 5% CO2_2_ incubator to eliminate extracellular bacteria. Subsequently, the cells were washed again with PBS and lysed with 1% Triton X-100. The lysates were serially diluted and plated on LB agar plates, which were incubated at 37°C for 16 h. The number of intracellular bacteria was quantified by counting colony-forming units (CFUs) on LB agar plates relative to the inoculum to calculate an invasion percentage.

### Immunofluorescence-based detection of bacterial invasion

Approximately 1 × 10^5^ Caco-2 cells were seeded onto collagen-coated coverslips in 6-well plates. On day 3 (or when > 90% confluent), the cells were washed with warm, antibiotic, and serum-free medium, replenished with fresh antibiotic and serum-free DMEM medium, and infected with *E. coli* strains NRG857c, NRG857c ∆*pduB*, and NRG 857C ∆*pdu*B + *pduB* at an MOI of 10. Following centrifugation at 500 × *g* for 15 min, the cells were incubated at 37°C for 3 h. After incubation, the cells were washed and incubated with serum-free DMEM containing 240 μg/mL amikacin for 1 h at 37°C to eliminate extracellular bacteria. The cells were then washed twice with PBS, fixed with 4% paraformaldehyde (PFA), and permeabilized with 0.1% Triton X-100. Cells were blocked with 1% bovine serum albumin (BSA) at room temperature for 1 h and incubated at 4°C with an anti-*E*. *coli* LPS antibody (rabbit polyclonal, Abcam AB35654), diluted 1:500 in 1% BSA. After three washes with PBST (PBS containing 0.1% Tween 20), the cells were incubated with a goat anti-rabbit Alexa Fluor 488 secondary antibody (Thermo Fisher A-11008), diluted 1:1000 in 1% BSA, for 1 h at RT. Cells were counterstained using rhodamine-phalloidin (Fisher Scientific R415) and mounted using medium containing DAPI. Images were acquired using a Leica laser scanning confocal microscope at 63× magnification. At least 10 high-power field (HPF) images were captured for each group, and intracellular bacteria and cells were counted.

### Measurement of bacterial growth curve

AIEC strains NRG857c and NRG857c ∆*pduB* were cultured in LB or DMEM medium for 12 h in 96-well plates. Bacterial growth was monitored by measuring the optical density at 600 nm (OD_600_) using a spectrophotometer.

### Quantification of bacterial biofilm formation

AIEC strains NRG857c and NRG857c ∆*pduB* were cultured in glass plates for 72 h at 37°C. On day 3, the medium was removed, and the plates were washed several times with tap water to eliminate non-adherent cells. A 0.1% crystal violet solution was then added to each plate and incubated at RT for 15 min. After staining, the plates were thoroughly washed with tap water to remove excess dye. Dimethyl sulfoxide (DMSO) was then added to solubilize the bound crystal violet, and the plates were incubated for an additional 15 min at RT. Then, 100 μL of the DMSO solution was collected from each sample in triplicate and transferred to a 96-well plate. Biofilm formation was quantified by measuring absorbance at 570 nm using a spectrophotometer.

### Quantification of fecal *E. coli* colonization in antibiotic-treated mice

*Il10^−/^*^−^ mice were treated with an antibiotic cocktail (metronidazole [1 g/L], neomycin [1 g/L], vancomycin [500 mg/L], and ampicillin [1 g/L]) via oral gavage (100 µL per mouse) for 3 consecutive days to deplete the gut microbiota, as described earlier (69). On day 3 of antibiotic treatment, fecal samples were collected and plated on LB agar to verify microbiota depletion. Forty-eight hours after the final antibiotic treatment, microbiota-depleted *Il10^−/^*^−^ mice were colonized with the NRG857c (1 × 10^8^ CFU per mouse) once daily for 3 consecutive days. From day 3 post-colonization onwards, fecal samples were collected and plated on LB agar to quantify bacterial CFUs.

### *In vivo* bacterial competition assay

Antibiotic-treated *Il10^−/^*^−^ mice (as described above) were colonized with a 1:1 mixture of wild-type *E. coli* NRG857c (ampicillin-resistant) and the isogenic Δ*pduB* mutant (engineered to carry kanamycin resistance). The wild-type NRG857c strain carries intrinsic ampicillin resistance (Amp^R^), whereas the Δ*pduB* mutant carries a kanamycin resistance cassette (Kan^R^). Each mouse received three consecutive daily oral gavages, with each gavage consisting of 1 × 10^8^ CFU of each strain per mouse (i.e., 1 × 10^8^ CFU WT + 1 × 10^8^ CFU Δ*pduB*; final 1:1 ratio in a total inoculum of 2 × 10^8^ CFU per mouse per day).

Fecal samples were collected on days 4, 8, 12, and 16, weighed, homogenized in sterile PBS, serially diluted, and plated on LB agar containing ampicillin or LB agar containing ampicillin plus kanamycin. Colonies recovered on ampicillin-containing plates represented the total bacterial population (wild-type NRG857c plus Δ*pduB*), whereas colonies recovered on ampicillin-plus-kanamycin plates represented the Δ*pduB* population. Wild-type NRG857c CFUs were calculated by subtracting Δ*pduB* CFUs from the total CFUs recovered on ampicillin plates.

To induce intestinal inflammation, mice were switched to a piroxicam-fortified diet on day 8. On day 18, mice were euthanized and colons were harvested. Colonic fecal contents were gently removed to eliminate luminal bacteria. The colonic mucus layer was carefully scraped and collected from the colon, and both the mucus and colonic tissues were weighed and homogenized separately in sterile PBS. Homogenates were serially diluted and plated on selective LB agar as described above. Following overnight incubation at 37°C, CFUs were counted. For each fecal, colonic mucus, and colonic tissue sample, bacterial competition was expressed as the log competitive index (log CI). A log CI value of 0 indicates equal representation of the two strains, whereas positive and negative values indicate a competitive advantage of wild-type NRG857c or the Δ*pduB* mutant, respectively. Luminal colonization was assessed using fecal log CI values, whereas mucosal association was evaluated using log CI values from colonic mucus and colonic tissue samples.

### Mouse model of AIEC-induced colitis using *Il10^−/^*^−^ mice

Antibiotic-treated 8- to 10-week-old *Il10^−/^*^−^ mice were colonized with NRG857c and NRG857c ∆*pduB*. To assess spontaneous colitis, body weight and rectal prolapse were monitored every other day. To induce colitis, mice were fed a piroxicam-fortified diet for 5 consecutive days. Piroxicam was purchased from Sigma-Aldrich (Catalog P0847), and the diet was prepared by Research Diets by mixing 200 ppm of piroxicam with regular chow. Body weight loss was monitored to assess piroxicam-induced colitis in the colonized mice.

### Histopathological evaluation of colon

Colons were collected aseptically, washed thoroughly with PBS, and fixed in 10% formalin for 48 h at RT. Fixed tissues were processed by the Histopathology Core at UT Southwestern Medical Center (UTSW), and H&E-stained images were captured at 4× and 10× magnifications using a Keyence microscope.

### RNA isolation and gene expression analysis by qRT-PCR

Whole colons were homogenized in TRIzol reagent (15596018, Invitrogen), and total RNA was isolated following the manufacturer’s protocol. RNA was reverse-transcribed into cDNA using the High-Capacity cDNA Reverse Transcription Kit (4368814, Applied Biosystems). qRT-PCR was performed using PowerTrack SYBR Green Master Mix (446109, Applied Biosystems) with gene-specific forward and reverse primers, as listed in Table S1. Gene expression data were normalized to the housekeeping gene Gapdh, as described previously (70, 71).

### Transepithelial electrical resistance and permeability assay

Caco-2 cells (1 × 10^5^) were seeded into collagen-coated 24-well Transwell plates (3421, Corning). On day 5 (or when >90% confluent), the cells were washed with warm, antibiotic-free medium, replenished with fresh antibiotic-free medium, and infected with AIEC strains NRG 857C, NRG 857C ∆*pduB*, and NRG 857C ∆*pduB + pduB* at an MOI of 10. Following centrifugation at 500 × *g* for 15 min, the cells were incubated at 37°C for 3 h. Following incubation, the cells were washed with pre-warmed medium and then incubated in DMEM containing 240 μg/mL amikacin for 1 h at 37°C to remove extracellular bacteria. The transepithelial electrical resistance (TEER) was measured using a voltohmmeter (Millicell ERS-2). After three washes with PBS, 100 μL of FITC-dextran (FD-4; Sigma) diluted in PBS (1 mg/mL or 100 μg/well) was added to the upper chamber of the Transwell and incubated for 20 min at 37°C. Following incubation, PBS from the lower chamber was collected, and relative fluorescence units (RFU) were measured using a plate reader (Gen 5) with excitation at 485 nm and emission at 535 nm. RFU values were calculated using a FITC-dextran standard curve.

### Measurement of vitamin B_12_ in blood serum

Blood was collected from mice and allowed to clot for at least 30 min at RT before centrifugation at 10,000 rpm for 10 min at 4°C. Serum was separated and stored at −80°C until vitamin B_12_ measurement using a vitamin B_12_ ELISA kit, according to the manufacturer’s instructions (ab287826, Abcam).

### Statistical analysis

Data are expressed as the mean ± standard error of the mean (SEM). Age- and sex-matched male and female mice were used in all experiments. For comparisons between two groups, 2-tailed Student’s *t-*tests were performed. For multi-group comparisons, one-way or two-way analysis of variance (ANOVA) was used. For bacterial competition experiments, Wilcoxon signed-rank tests were used to determine whether log CI values differed significantly from zero. Statistical significance was defined as a *P* value of <0.05. All statistical analyses were performed using GraphPad Prism 10 software.

## Data Availability

All data generated or analyzed during this study, including supporting data, are available from the corresponding author upon reasonable request.

## SUPPLEMENTAL MATERIAL

Supplemental material mbio.01698-26-s0001.pdf Supplemental figures and table.

ASM does not own the copyrights to Supplemental Material that may be linked to, or accessed through, an article. The authors have granted ASM a non-exclusive, world-wide license to publish the Supplemental Material files. Please contact the corresponding author directly for reuse.
