# Supplementary material for: Adherent-invasive Escherichia coli pduB drives vitamin B12-dependent colitis in mice
Source: mBio. 2026 Sep 16;17(10):e01698-26. doi: 10.1128/mbio.01698-26 (PMC13712824; doi:10.1128/mbio.01698-26)
Supplement: Supplementary file 1 [file mbio.01698-26-s0001.pdf]

Supplemental data

Adherent-invasive *Escherichia coli pduB* drives vitamin B_{12}-dependent colitis in mice

Shahanshah Khan[1], Jungyeon Kim[1], Alexander Lafrance[1], Sydney Field[1,2], Xi Li[1], Lisa N. Kinch[2], Kevin J. Forsberg[3], Tamia Harris-Tryon[4], Qian Cong[5,6,7], Kim Orth[2,8,9], Hans-Christian Reinecker[1], and Josephine Ni[1,3]

[1]Division of Digestive and Liver Diseases, Department of Internal Medicine, University of Texas Southwestern Medical Center, Dallas, Texas-75390, USA

[2]Department of Molecular Biology, University of Texas Southwestern Medical Center, Dallas, Texas-75390, USA

[3]Department of Microbiology, University of Texas Southwestern Medical Center, Dallas, Texas-75390, USA

[4]Department of Dermatology, University of Texas Southwestern Medical Center, Dallas, Texas-75390, USA

[5]Eugene McDermott Center for Human Growth and Development, University of Texas Southwestern Medical Center, Dallas, Texas-75390, USA

[6]Department of Biophysics, University of Texas Southwestern Medical Center, Dallas, Texas-75390, USA

[7]Harold C. Simmons Comprehensive Cancer Center, University of Texas Southwestern Medical Center, Dallas, Texas-75390, USA

[8]Howard Hughes Medical Institute, University of Texas Southwestern Medical Center, Dallas, Texas-75390, USA

[9]Department of Biochemistry, University of Texas Southwestern Medical Center, Dallas, Texas-75390, USA

Corresponding Author

Josephine Ni, M.D.
Division of Digestive and Liver Diseases,
Department of Internal Medicine,
University of Texas Southwestern Medical Center,
Dallas, Texas-75390-9195, USA
Phone No.: +1-214-645-9587
Email: Josephine.Ni@UTSouthwestern.edu

Supplemental figure with its legend

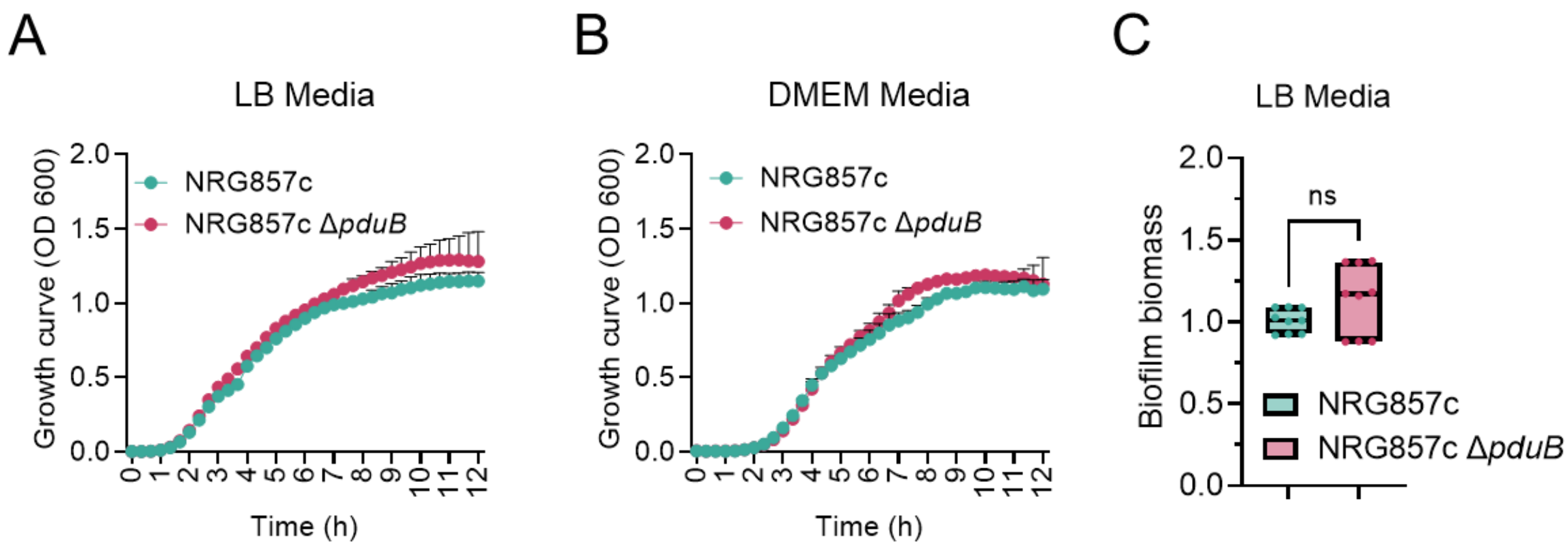

Figure S1. *pduB* gene deletion does not affect *E. coli* growth and biofilm formation in NRG857c. (A-B) AIEC strains NRG857c and NRG857c Δ*pduB* were cultured in either LB or DMEM medium for 12 hours in a 96-well plate. Growth was monitored by measuring optical density at 600 nm (OD_{600}) using a plate reader. (C) AIEC strains NRG857c and NRG857c Δ*pduB* were cultured in glass plates for 72 hours at 37°C. On day 3, the media was removed, and the plates were washed several times to eliminate non-adherent cells. A 0.1% crystal violet solution was then added to each plate and incubated at room temperature (RT). After staining, dimethyl sulfoxide (DMSO) was added to solubilize the bound crystal violet, and the absorbance at 570 nm was measured using a spectrophotometer to quantify biofilm formation. Statistical significance at the final timepoint was assessed using an unpaired 2-tailed Student's t test. Comparisons involving only two groups were analyzed using an unpaired 2-tailed Student's t test.

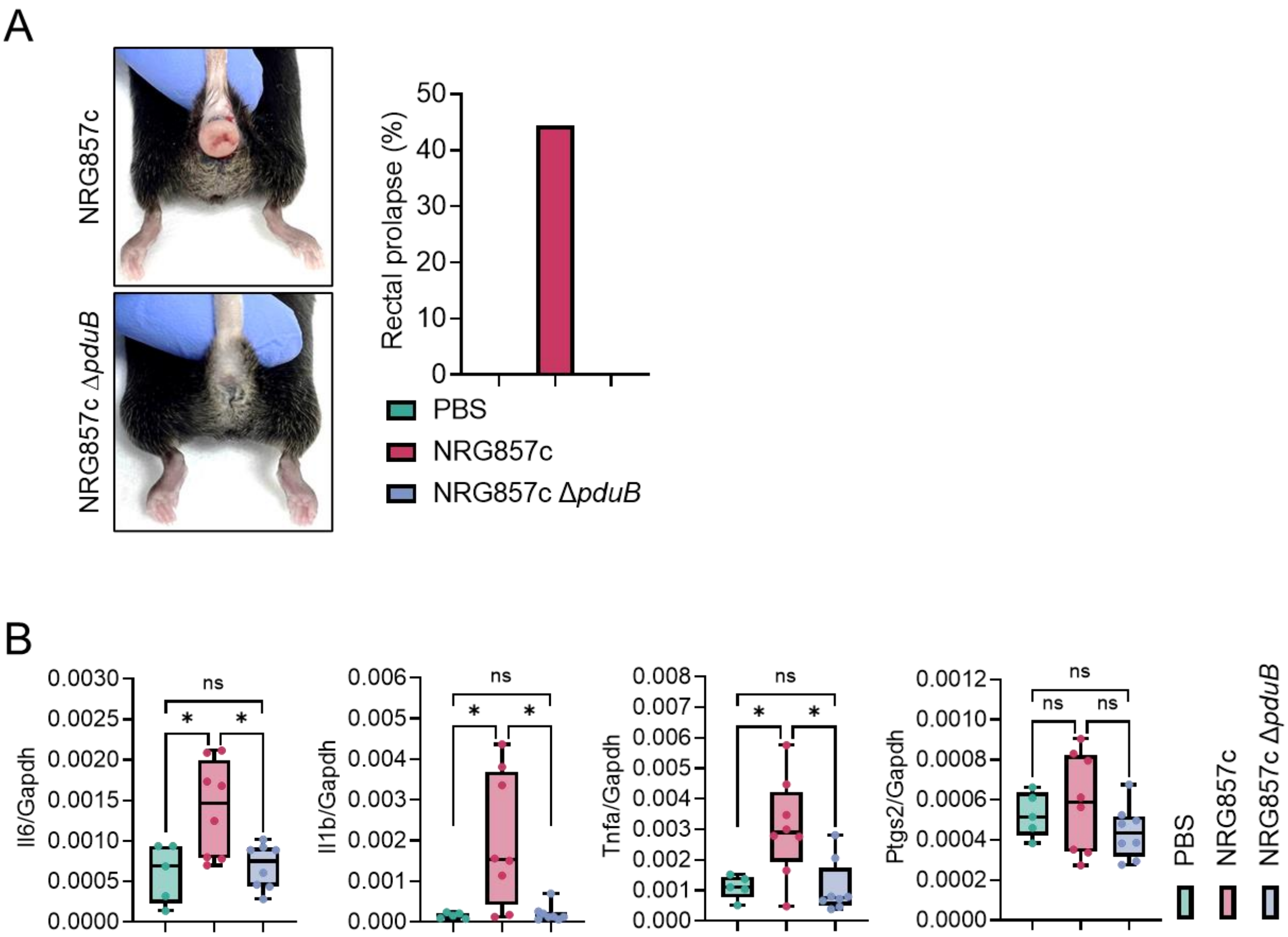

Figure S2. *pduB*-deficient NRG857c strain suppresses inflammatory responses in *Il10*$^{-/-}$ mice. Antibiotic-treated mice were colonized with NRG857c or NRG857c Δ*pduB*. (A) Rectal prolapse was assessed on day 26. (B) On day 26, mice were euthanized, and colons were collected. Colons were homogenized in TRIzol to isolate RNA, which was used to measure the gene expression levels of *Il6*, *Il1b*, *Tnfa*, and *Ptgs2*. *Gapdh* was used as a housekeeping gene. One-way ANOVA was used for comparisons involving multiple groups. Comparisons involving only two groups were analyzed using an unpaired 2-tailed Student's t test.

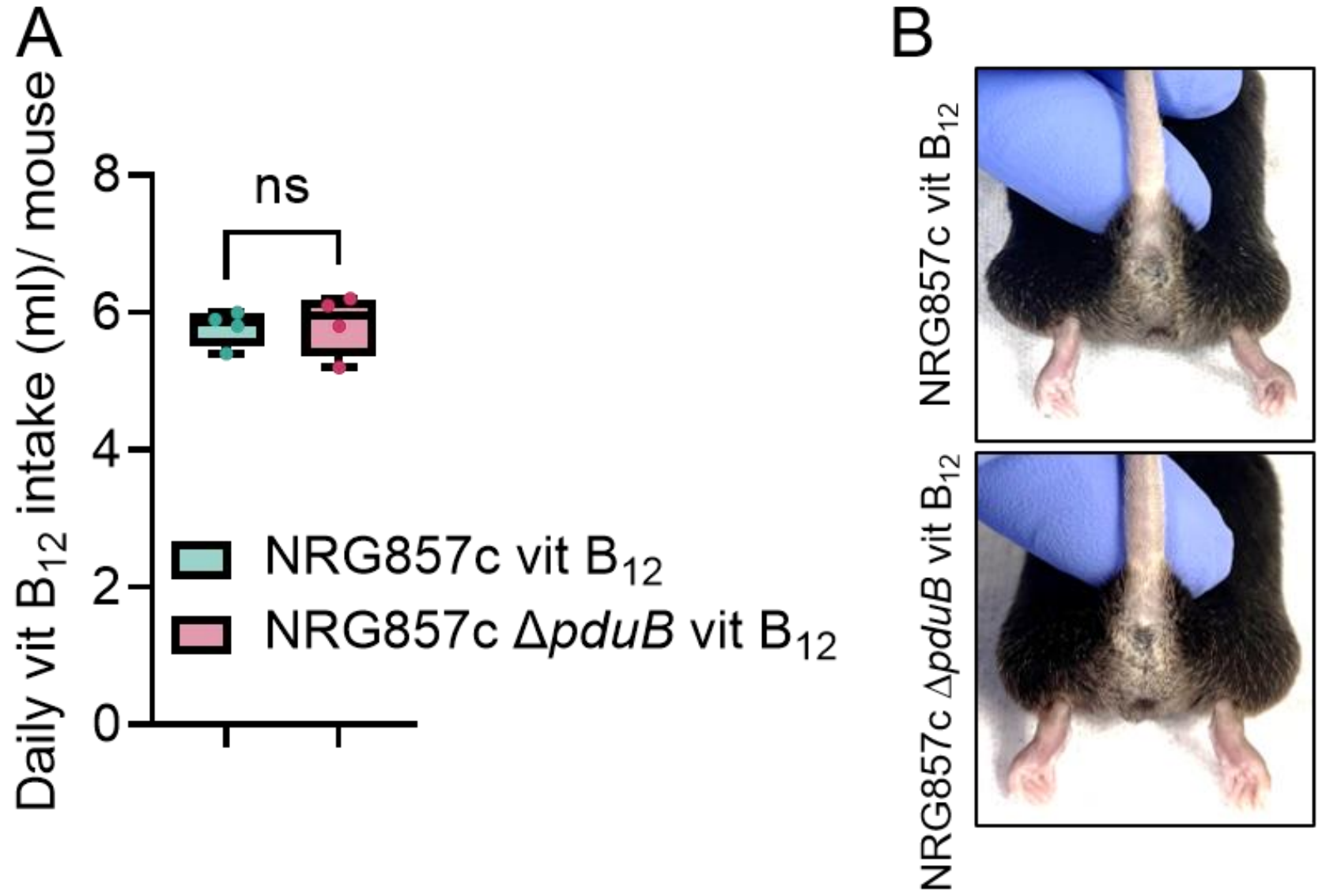

Figure S3. Protective role of vitamin B_{12} in *Il10*$^{-/-}$ colonized with NRG857c. Antibiotic-treated mice were colonized with NRG857c or NRG857c Δ*pduB*. (A) Starting on day 4 post-colonization, mice were administered vitamin B_{12} in their drinking water (*ad libitum*). (B) Rectal prolapse was assessed on day 26. Comparisons involving only two groups were analyzed using an unpaired 2-tailed Student's t test.

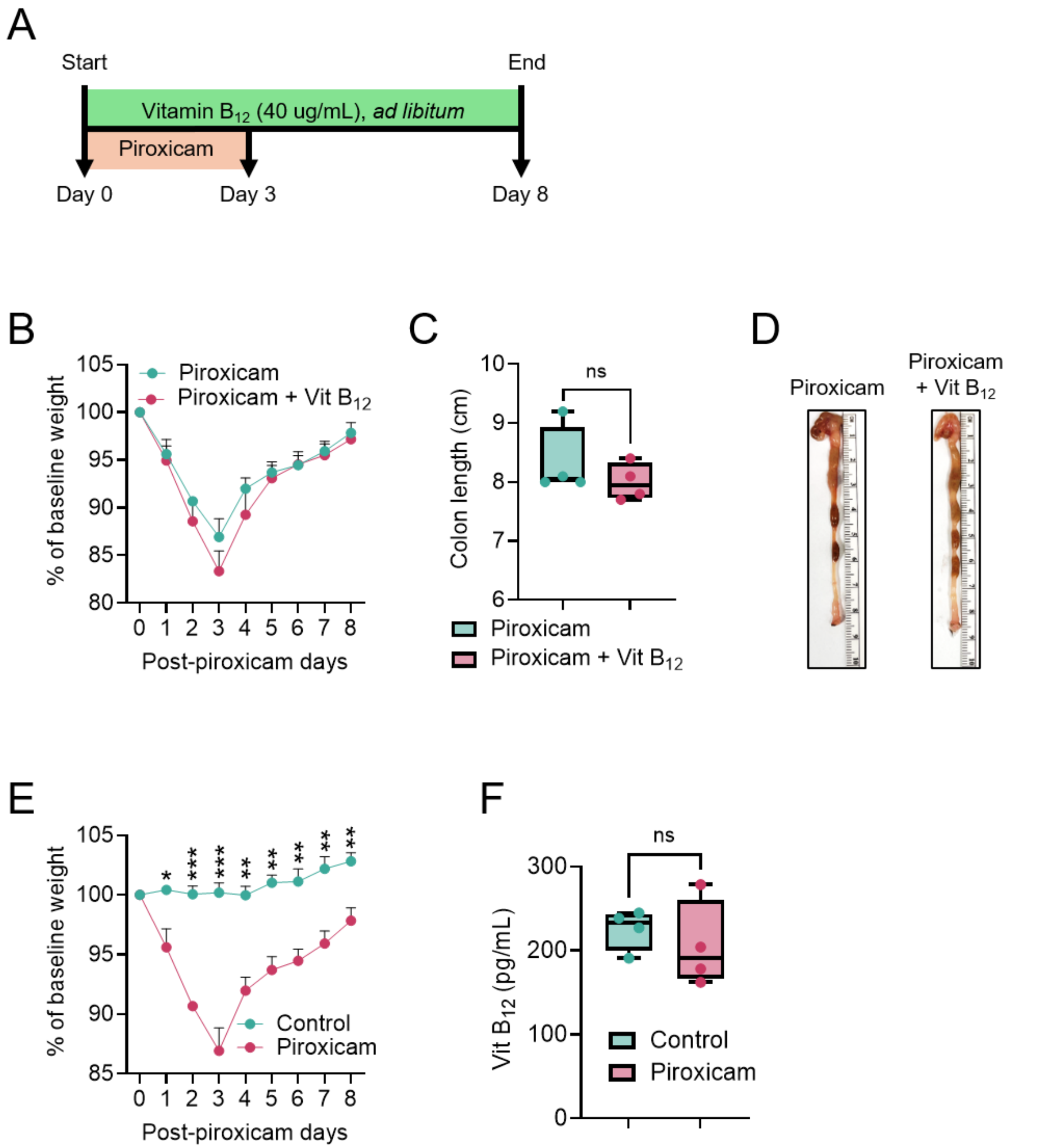

Figure S4. Vitamin B_{12} does not suppress piroxicam-induced colitis or alter systemic vitamin B_{12} levels during intestinal inflammation in *Il10*$^{-/-}$ mice. (A) Experimental design of vitamin B12 supplementation in the piroxicam-induced colitis model. Starting on day 0, piroxicam-fed *Il10*$^{-/-}$ mice were provided vitamin B_{12} in drinking water *ad libitum*. (B) Body weight changes were recorded throughout the experiment. (C-D) On day 8, mice were sacrificed and colon length was measured. (E-F) *Il10*$^{-/-}$ mice were fed with a piroxicam-fortified diet to induce colitis. Body weight changes were monitored over time following piroxicam administration (E). On day 8 following piroxicam treatment, mice were sacrificed and vitamin B_{12} levels (in serum) were measured using an ELISA kit (F). Statistical significance was determined using two-way ANOVA and unpaired two-tailed Student's t test.

Table S1. List of Primers

Primer	Sequence (5`-3`)	Reference
pduB F	CCCCGCCCACATACCGATGTCGAAAAAATCTTACCAAAGGGA ATTAACCCCTGTCAAACATGAGAATTAA	This study
pduB R	TTTGCCAGTGCTTCAAATCTTTTCGATCTCATAAATCTGCCTCA GAGAATGTGTAGGCTGGAGCTGCTTC	This study
Il6 F	CAA GAA AGA CAA AGC CAG AGT C	70
Il6 R	GAA ATT GGG GTA GGA AGG AC	70
Il1b F	GCCTCGTGCTGTCGGACCCATA	70
Il1b R	TGCAGGGTGGGTGTGCCGTCTT	70
Tnfa F	TCCCAGGTTCTCTTCAAGGGA	70
Tnfa R	GGTGAGGAGCACGTAGTCGG	70
Ptgs2 F	CCAGAGCAGAGAGATGAA	69
Ptgs2 R	GGTACAGTTCCATGACATC	69
Gapdh F	TGG CAA AGT GGA GAT TGT TGC C	70
Gapdh R	AAG ATG GTG ATG GGC TTC CCG	70
